# Reviewers Acknowledgement

**DOI:** 10.1002/1878-0261.70219

**Published:** 2026-02-25

The Editors of *Molecular Oncology* would like to thank all referees who have given their time and expertise to review articles submitted for publication in Volume 19. *Molecular Oncology* heavily relies on reviewers' careful assessment, constructive criticism, and timely response to ensure both quality and fast turnaround times.

The names of these reviewers are listed below; we apologize if we have inadvertently omitted anyone.

Afshar Saeid

Ahmad Mohammad

Albrengues Jean

Ali Simak

Almonte Maribel

Aloyz Raquel

Al‐Shaheri Fawaz N.

Amantini Consuelo

Ammeling Jonas

Amodio Nicola

Andersson Tommy

Andolino Chaylen

Appleton Lizzie

Arechage‐Ocampo Elena

Ashur‐Fabian Osnat

Ávila Matías Antonio

Awah Chidiebere

Ayoub Nabieh

Ayuda‐Durán Pilar

Baas Paul

Baba Masaya

Baker Kristi

Bakht Martin

Balasubramanian Poonkuzhali

Balic Marija

Balliu Manjola

Barker Holly E.

Barreto Guillermo

Barron Tara

Bartek Jiri

Bartelink Imke Heleen

Barzegar Behrooz Amir

Basrai Munira

Basu Abhijit

Baughn Linda

Baxter Robert C.

Becker Dorothea

Benitez Riquelme Diego

Bergholtz Helga

Bijlsma Maarten

Birkbak Nicolai Juul

Blanquart Christophe

Blattmann Peter

Blazek Dalibor

Blomme Arnaud

Blyth Karen

Bocci Matteo

Bommireddy Ramireddy

Bonavida Benjamin

Borkowska Edyta

Borriello Lucia

Bost Frédéric

Boucai Laura

Boulagnon‐Rombi Camille

Brandalise Federico

Braun Marcin

Brennen W. Nathaniel

Brix Nikko

Brodeur Melica Nourmoussavi

Brown Jennifer R.

Bu Lin‐Lin

Bulstrode Harry

Bussolino Federico

Bustos Matias

Caini Saverio

Campaner Stefano

Caraglia Michele

Caratti Bozhena

Carbone Carmine

Carrer Alessandro

Carrera Ramírez Ana Clara

Casagrande Giovanna Maria Stanfoca

Casini Angela

Cavazzoni Andrea

Chang Ying‐Jun

Chaveroux Cedric

Chen BaoQing

Chen Danyu

Chen Ming

Chen Weikang

Chen Wenan

Chen Yinnan

Cheresh David

Chu Simon

Cifani Paolo

Cingöz Ahmet

Clark Amanda

Classens F.

Clemons Nicholas

Clynes Martin

Coker‐Gurkan Ajda

Colnot Sabine

Condello Vincenzo

Cong Bojie

Cook Leah

Cooper Wendy

Cordero Julia

Correia Ana Luisa

Coskun Ahmet

Cottard Félicie

Cox Thomas

Cristofari Gael

Crocetto Felice

Culig Zoran

Cusan Monica

Dahlhoff Maik

Damia Giovanna

Dange Riya

Daubon Thomas

De Antonellis Pasqualino

de Bock Charley

de Cárcer Guillermo

de Jong Steven

De los Santos Mireia Cruz

De Rosa Viviana

De Zio Daniela

Debnath Jayanta

DeBono Johann

De Maria Raffaella

Deuter Max

Devarajan Raman

DeWitt Tyler

Diaferia Giuseppe Riccardo

Diefenbach Russell

Dieguez Lorena

Dika Emi

Dimopoulos Konstantinos

Ding Daisy Xiao

Ding Yanqiang

Dopeso Higinio

Dragomir Mihnea P.

Drapela Stanislav

Driscoll James

Drosten Matthias

Duarte Delfim

Eadie Laura N.

Edeline Julien

Edmondson Elijah F.

Eickhoff Nils

Eilers Martin

Eiras Mariana

Eiring Anna M.

Eisenman Robert

El Yazidi‐Belkoura Ikram

Endo M.

Erlacher Miriam

Erson‐Bensan Ayse Elif

Estella Carlos

Eyers Patrick

Fagereng Gro Live

Fang Wenfeng

Färkkilä Anniina

Fasken Milo

Felley‐Bosco Emanuela

Felsenstein Matthäus

Fenton Tim

Fiedler W.

Fischer Andreas

Foidart Pierre

Foster Leonard

Fre Silvia

Freeman Kevin

Friis Soren

Fritz Connor

Furukawa Natsuki

Gabrilovich Dmitry I.

Galante Pedro A.F.

Gammoh Noor

Ganley Ian G.

Gao Zhengliang

Garcia Reynaldo L.

Garcion Emmanuel

Gardiner Jaye C.

Gareen Ilana

Garikipati Venkata Naga Srikanth

Garzoni Luciana Ribeiro

Gately Kathy

Giacomelli Chiara

Giacomelli Luca

Giacomini Patrizio

Gkretsi Vasiliki

Golubnitschaja Olga

Gorgoulis Vasilis

Grassi Elena

Grauslund Morten

Greene Mark I.

Grünewald Thomas

Guerrier Camille

Guida Florence

Guikema Jeroen

Gumulec Jaromir

Guney Eskiler Gamze

Guo Weijian

Gupta Avantika

Gupta Nishma

Haemmerich Dieter Georg

Hamdy Nadia M.

Hao Xingjie

Harada Hiroshi

Haricharan Svasti

Hartman Mariusz Lukasz

Hatakeyama Keiichi

Hayashi Takuma

He Ling

He Xueyan

He Yaowu

Heckman Caroline A.

Hegi Monika

Helleday Thomas

Hermawan Adam

Hernandez‐Ramirez Laura C.

Hernández‐Rivas Jesús María

Hernando Barbara

Herrero Rolando

Hoare Matthew

Hodny Zdenek

Hofman Paul

Horiuchi Yutaka

Hou Jian

Huang Yizhou

Hühn Daniela

Hultstrand Cecilia

Hurwitz Stephanie N.

Ibarra Luis Exequiel

Igelmann Sebastian

Illi Barbara

Indra Arup Kumar

Iqbal Jabed

Itahana Koji

Iwakuma Tomoo

Jackson Sadhana

Jagadeeshan Sankar

Jalkanen Katriina

Janik‐Koncewicz Kinga

Jenab Mazda

Jeronimo Carmen

Johnson Rory

Jolly Mohit Kumar

Ju Jing Fang

Junier Marie‐Pierre

Justicia Lirio Pedro Luis

Kallergi Galatea

Kampen Kim

Kampman Ellen

Kanamarlapudi Venkateswarlu

Kane Lawrence P.

Kao Shao‐Hsuan

Karadimitris Anastasios

Karamanos Nikos

Kasimir‐Bauer Sabine

Keane Lily

Keppler Oliver

Kermorgant Stephanie

Kietzmann Thomas

Kim Chi‐Ju

Kim Hak Kyun

Kim Jaehoon

Kliment Corrine

Knight Katherine L.

Koguchi Yoshinobu

Kollmar Otto

Kondo Nobuyuki

Kondo Tadashi

Kontopodis Emmanouil

Korber Verena

Kortum Robert L.

Kos Lidia

Koskinen Paivi J.

Koti Madhuri

Kotula Leszek

Kowalik Artur

Koyasu Sho

Krejci Lumír

Kringelbach Tina

Kuhlmann Jan Dominik

Kumar Avinash

Kumar Ramesh

Kumar Sandeep

Lam W.K. Jacky

Lau Harry

Lavigne Pierre

Lazo John Stephen

Le Calvez‐Kelm Florence

Leask Andrew

Lecona Emilio

Lefebvre Tony Daniel

Lefrançois Philippe

Li Yan

Liang Shikang

Liang Yinwen

Lianidou Evi

Lim Chinten James

Lima Vladmir Claudio Cordeiro de

Lin Yufeng

Linder Stig

Lindquist Jonathan A.

Liou Angela

Liu Hsiao‐Sheng

Liu Miao

Liu Yali

Llanos Susana

Lohse Stefan

Lokeshwar Bal L.

Lonsdale Andrew

López‐Pernas Gema

Lord Christopher

Lu Xiaofan

Lu Yinghong

Ma Haowei

Maass Kendra

Madapura Pradeep

Maeda Shina

Makvandi Manoochehr

Malapelle Umberto

Malecka‐Wojciesko Ewa

Malla Saloni

Malli Naniye

Marasca Federica

Martinez Salvador

Martin‐Guerrero Idoia

Marzo Isabel

Mateus Pedro

Matta Michele

Mazitschek Ralph

McBride William

Medina Heidy N.

Medova Michaela

Meiser Johannes

Menga Alessio

Merlini Alessandra

Micol Jean‐Baptiste

Mieczkowski Jakub

Miękus Katarzyna

Miele Evelina

Mishra Avanish

Mitra Suman

Modesti Mauro

Molina‐Vila Miguel A.

Mondal Jayanta

Montfort Anne

Moradi Arash

Moreaux Jerome

Moresi Viviana

Moretti Fabiola

Moss Esther

Moura‐Neto Vivaldo

Muiños Ferran

Murillo Raul

Nagasaka Takeshi

Nathan James A.

Nguyen Long

Niechi Ignacio

Nielsen Anders Lad

Nifantiev Nikolay E.

Nordgard Oddmund

Norris Lucy A.

Ntzifa Aliki

Ødum Niels

Ohara Yuuki

Ohki Rieko

Oliaro Jane

Olsson Ann

Ortmann Brian

Paccez Juliano

Padmanabhan Jaya

Panzuto Francesco

Papaccio Federica

Papakonstantinou Dimitrios

Park Jin Young

Paronetto Maria Paola

Pascal John M.

Pastor Pareja José Carlos

Pasupulati Anil Kumar

Patel Rajinikant

Pavlova Natasha

Peglion Florent

Peinelt Christine

Pellat Deceunynck Catherine

Pendas Alberto

Pentimalli Tancredi Massimo

Perez‐Chacon Gema

Peschiaroli Angelo

Peters Christian J.

Peuhu Emilia

Phillips Phoebe

Piaggio Giulia

Piekorz Roland

Pieraccioli Marco

Pierga Jean‐Yves

Pietras Kristian

Pineau Pascal

Pirlog Radu

Pisani Francesco

Pisapia Pasquale

Polasky Daniel A.

Poliseno Laura

Prado‐Garcia Heriberto

Prekovic Stefan

Pucci Perla

Puhka Maija

Puliafito Alberto

Pulvirenti Alfredo

Pusch Stefan

Qian Lilin

Rai Vikas

Ramos Kenneth S.

Redmer Torben

Reduzzi Carolina

Reed Damon R.

Rehwinkel Jan

Renneville Aline

Ricciardelli Carmela

Rieke Damian

Riese David

Riethdorf Sabine

Riggins Rebecca B.

Robinson Stephen

Rogatto Silvia Regina

Rohm Maria

Rosenfeld Michael Geoffrey

Rossi Tania

Rota Rosella

Rovida Elisabetta

Rowe Helen

Roy Ananda L.

Roz Luca

Ruff Samantha

Rutkowska Aleksandra

Sabarinathan Radhakrishnan

Sadler Edepli Kirsten C.

Sadras Teresa

Salmena Leonardo

Salomoni Paolo

Samoylenko Anatoliy

Sanchez‐Prieto Ricardo

Santana Codina Naiara

Santocanale Corrado

Santoni‐Rugiu Eric

Sartori Alessandro A.

Scala Stefania

Schlicke Pirmin

Schmalfeldt Barbara

Scholz‐Kreisel Peter

Schreml Stephan

Schumacker Paul T.

Scurll Joshua Mark

Secrier Maria

Selth Luke A.

Senturk Serif

Seo Sang Uk

Seoane Jose Antonio

Sharlow Elizabeth

Sharp Adam

Sharp Lena

Shaw Jacqui

Shifman Julia

Shomron Noam

Siddharth Sumit

Singh Abhishek Kumar

Singh Kanhaiya

Singhal Mahak

Slade Neda

Smerdou Cristian

Smits Veronique A.J.

Smythe Carl

Soni Aashish

Soucek Pavel

Spick Matt

Stathopoulos Georgios T.

Steenbergen Renske

Stoecklein Nikolas H.

Strand Trond‐Eirik

Sullo Francesco

Sun Lifeng

Szmajda‐Krygier Dagmara

Tadesse Solomon

Tagliabue Elda

Tan Kezhe

Tanaka Mamoru

Tejedor Juan Ramon

Telleria Carlos M.

Tellier Michael

Teng Yong

Terada Naoki

Termini Christina

Terp Mikkel

Theodorou Alexis

Tirado Oscar M.

Tirosh Amit

Togawa Kayo

Toledo Rodrigo A.

Torre Vincent

Tress Michael

Triulzi Tiziana

Tschurtschenthaler Markus

Tsukamoto Ichiro

Tulasne David

Turkoz Yusuf

Uhlmann Erik

Unione Luca

Unoki Motoko

Urbanska Edyta M.

Urda García Beatriz

Vaghasia Ajay

Valdivia Alejandra

Valdor Rut

van der Bruggen Pierre

Van Deventer Darcy

Van Doorn Remco

Vandeweyer Geert

Vega Rubin de Celis Silvia

Venuti Aldo

Veth Tim

Victori Pedro

Viktorsson Kristina

Vineis Paolo

Vinod Vivek

Virga Federico

Visal Tanvi

Vlachostergios Panagiotis J.

von Bubnoff Nikolas

von Kriegsheim Alexander

Voon Dominic Chih‐Cheng

Vorobyeva Anzhelika

Wahnou Hicham

Wandall Hans H.

Wang Lu

Wang Xinzheng

Watanabe Satoshi

Wei Andrew

Wei Dong‐Qing

Wei Gong‐Hong

Wei Xinzhao

Wellbrock Claudia

Westermarck Jukka

White Elizabeth A.

Wilhelm Imola

Wilson Timothy Richard

Wiman Klas G.

Winter Christof

Wollschläger Daniel

Wu Mengrui

Wu Qihui

Wuestefeldt Torsten

Wurm Alexander

Wyatt Alexander

Xu Kang

Xu Lixia

Yamamoto Yusuke

Yang Hongbin

Yang Pan‐Chyr

Yaniv Dan

Yelamos Jose

Ylstra Bauke

Yool Andrea

Yoshida Hiroshi

Yuan Zuofei

Zarrine‐Afsar Arash

Zebisch Armin

Zeljic Katarina

Zhang Jun

Zhang Ruyang

Zhang Shixiong

Zheng Qinheng

Zhou Bin‐Bing S.

Zhou Heming

Zhou Lei

Zhou Zhiwei

Zhu Shouhai

Zou Yutian

